# Mining version history to predict the class instability

**DOI:** 10.1371/journal.pone.0221780

**Published:** 2019-09-16

**Authors:** Shahid Hussain, Humaira Afzal, Muhammad Rafiq Mufti, Muhammad Imran, Amjad Ali, Bashir Ahmad

**Affiliations:** 1 Department of Computer Science, COMSATS University, Islamabad, Pakistan; 2 Department of Computer Science, Bahauddin Zakariya University, Multan, Pakistan; 3 Department of Computer Science, COMSATS University, Vehari, Pakistan; 4 Department of Computer, University of Swat, Swat, Pakistan; 5 Department of Computer Science, Qurtaba University, DIK, Pakistan; UCLA, UNITED STATES

## Abstract

While most of the existing class stability assessors just rely on structural information retrieved from a desired source code snapshot. However, class stability is intrinsically characterized by the evolution of a number of dependencies and change propagation factors which aid to promote the ripple effect. Identification of classes prone to ripple effect (instable classes) through mining the version history of change propagation factors can aid developers to reduce the efforts needed to maintain and evolve the system. We propose Historical Information for Class Stability Prediction (HICSP), an approach to exploit change history information to predict the instable classes based on its correlation with change propagation factors. Subsequently, we performed two empirical studies. In the first study, we evaluate the HICSP on the version history of 10 open source projects. Subsequently, in the second replicated study, we evaluate the effectiveness of HICSP by tuning the parameters of its stability assessors. We observed the 4 to 16 percent improvement in term of F-measure value to predict the instable classes through HICSP as compared to existing class stability assessors. The promising results indicate that HICSP is able to identify instable classes and can aid developers in their decision making.

## Introduction

The prediction of any software module which is more sensitive to changes in the future versions can aid software maintainer to reduce the maintenance time and efforts. According to ISO-9126, the maintainability of modules prone to changes in the system is defined as “software quality characteristic concerning the effort needed to make specified modifications to an already implemented system”, and characterized as analyzability, changeability, testability, and stability. In this article, we focused on the term stability (the opposite term is instability), which is defined as “characterizes the sensitivity to change of a given system that is the negative impact that may be caused by system changes”. The researchers have concentrated on change proneness at the certain granularity of software such as attribute, statement, method, class and file in several studies, and investigated the change impact by applying different techniques. In a recent review study, B. Li, et al. [1] have investigated 23 Change Impact Analysis (CIA) techniques and group them as 1) the techniques which based on the analysis of information collected during the execution of a program, 2) the techniques which based on information mined from the software repositories, 3) the techniques which based on different types of coupling such as structural, conceptual, dynamic function, and relational, and 4) the techniques using hybrid approach by combining several change impact analysis techniques. Usually, changes in a class yield in the response of 1) adding new requirements, 2) to perform the debugging activities, and 3) propagated changes occur in other classes [2, 3, 4]. The change proneness referred as the measurement of all these changes while the stability only referred as the measurement of propagating changes occurs in other classes. Ampatzoglou et al. [5] define and measure the term instability (opposite of term stability) as “the degree to which a class is subject to change, due to changes in other related classes andconsidering the probability of such classes to change as equal to a certain value”. Subsequently, the authors reported that the instable classes are more prone to the ripple effect promoted due to change propagation factors.

The researcher’s efforts are made in two aspects to assess whether a class is prone to ripple effect or not, 1) the probability of the source class undergoes a change is assessed by analyzing the source code change history, and 2) the dependencies that aid to propagate the changes to dependent classes and can be assess through potential metrics used in structural analysis. Generally, the coupling metrics are used to assess the structural complexity;. However, these coupling metrics cannot assess the class prone to the ripple effect [6]. In a recent study, Arvanitou et al. [7] describe a structural coupling metric named Ripple Effect Measure (REM) by estimating the number of dependencies and the probability of propagated change through these dependencies (i.e. Propagation factors). Subsequently, authors validate the capacity of REM as an assessor to estimate the probability of a class prone to the ripple effect.

**Fig 1:**
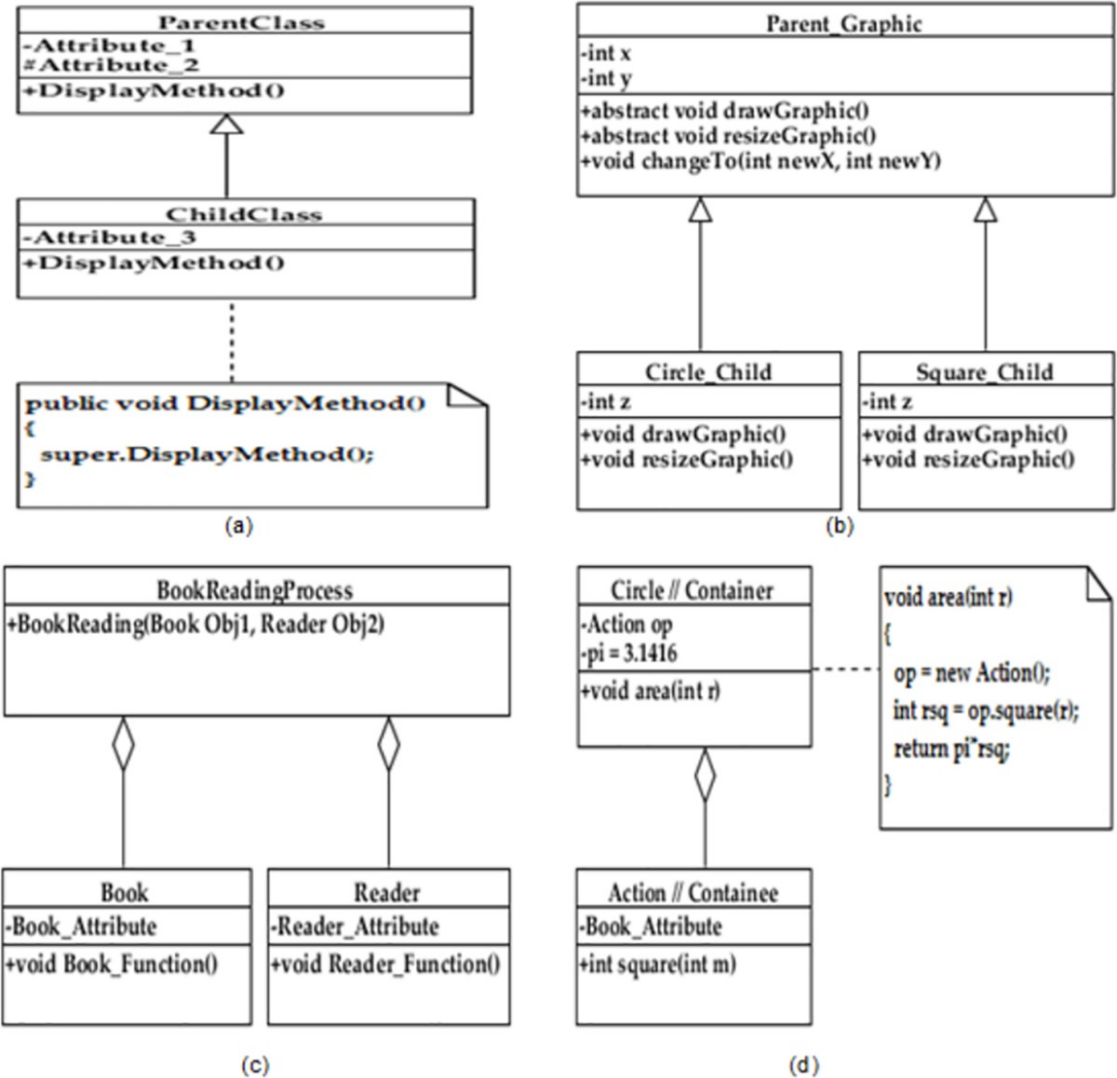
Realization of change propagation factors. a) Super method call, b) Overridden of abstract methods, c) Object creation as parameters of method of a container class d). Object creation in realization of method of a container class.

There are three types of class dependencies namely containment, generalization, and association [2], which can aid to evaluate the correlation between propagation factors and class instability. The containment dependency describe the “has-a” relationship which can propagate changes due to method calls of the container class to the public interface of the containee class (Fig 1C and 1D). The generalization dependency describe the “is-a” relationship and change propagation occur due to 1) access of protected attributes, 2) invocation of super methods and 3) overridden of abstract methods by a subclass (Fig 1A and 1B). The association type promotes the change propagation due to method calls from a class to another class through its public interface. In Fig 1, these propagation factors are formulated. For example, in Fig 1A, the stability of ‘ParentClass’ class depends on the realization of its method namely ‘DisplayMethod’ using the super method call in the derived class named ‘ChildClass’. The impact of this change propagation factor (i.e. Super method call) might be evolved in the subsequent releases due to increase in the number of derived classes. Consequently, we can conclude that the relationships among classes are considered as axes of change which can propagate changes from source classes to their dependent classes.

There exists a number of coupling metrics [8, 9, 10, 11], which have been validated both theoretically and empirically to assess the class stability. For example, Coupling Between Object (CBO) and Response For a Class (RFC) from Chidamber and Kemerer’s metrics suit [9, 11], Data Abstraction Coupling (DAC) and Measure of Aggregation (MOA) from QMOOD [10], Message Passing Coupling (MPC) [12] and REM [6]. These coupling metrics are based on method invocation and attributes references [13]. While the existing metrics exhibit good prediction of class stability by characterizing the source code snapshots. However, they still might not be adequate to capture the historical information about change propagation factors, including other factors (such as modification due to corrective maintenance) in order to predict the instable classes.

**Fig 2:**
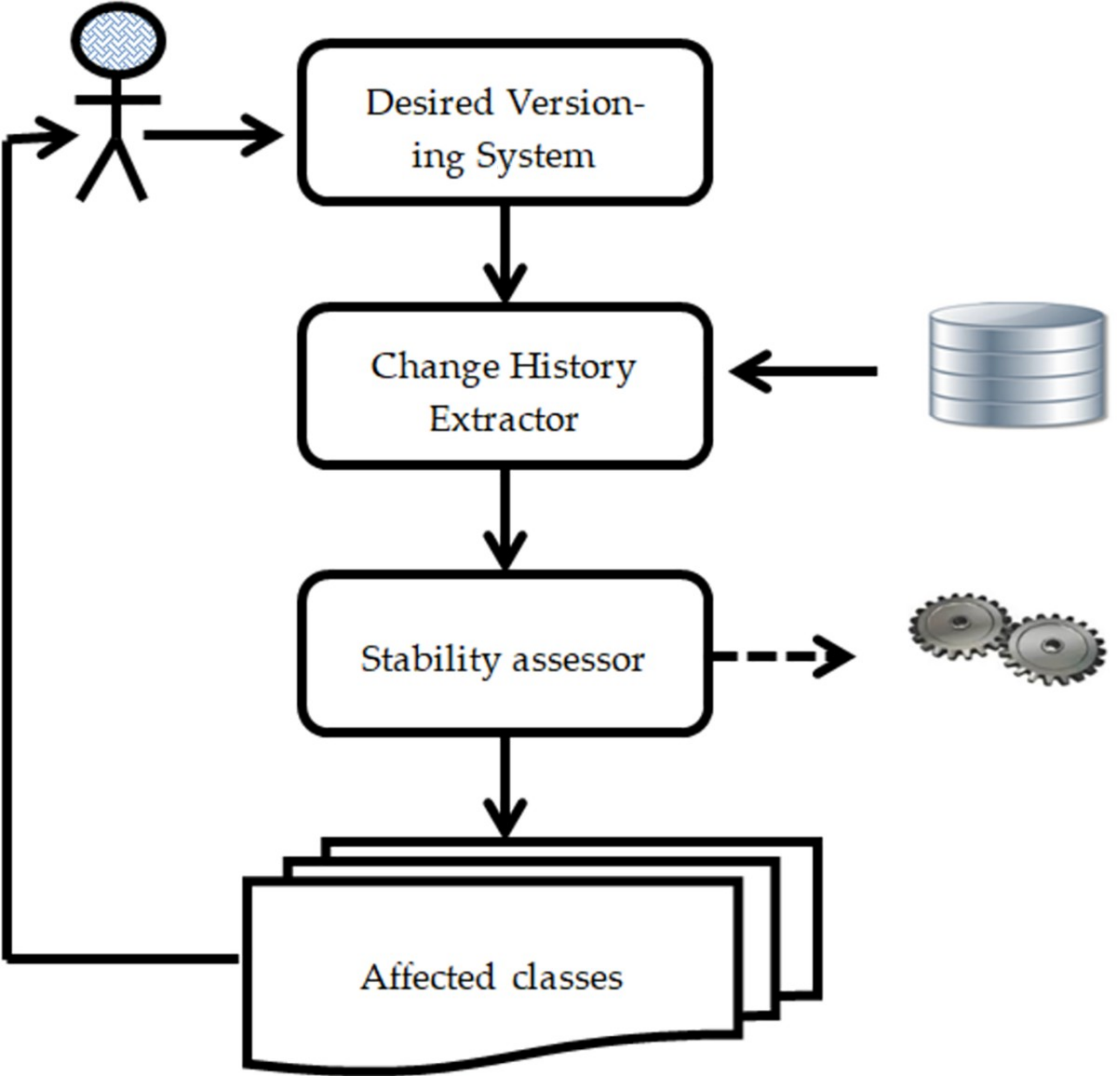
Overview of HICSP.

In order to address this issue, we propose an approach namely Historical Information for Class Stability Prediction (HICSP) to predict the instable classes based on the historical information about change propagation factors mined from the versioning systems. The framework of HICSP is described in Fig 2 and discussed in Section 3. In this paper, we perform two studies for the empirical investigation of the HICSP. In the first study, we performed experiments (with the same procedure) to evaluate the applicability and effectiveness of HICSP as compared to existing stability assessors on version history of 10 open source projects using widely-used evaluation criteria. The descriptive statistics about projects (under study) are shown in Table 1. Subsequently, in the second replicated study, we empirically investigate the effect of parameters tuning on the performance of HICSP with the version history of an open source project named MongoDB Java driver. The significant results of both studies present the effectiveness and applicability of proposed approach HICSP in order to identify the instable classes.

**Table 1:** Descriptive statistics of projects.

Project	artifactID	Time Slot	Total Snapshots	Classes in Snapshot s
MongoDB Java Driver	mongo-Java-driver	Oct, 2009 to Apr, 2016	88	268
Apache CXF	cxf-xerces-xsd-validation	Apr, 2009 to Oct, 2015	83	976
Apache Lucene	lucene-memory	Jun, 2007 to Apr, 2016	59	20
Google Guava	guava	Apr, 2010 to Dec, 2015	43	469
Apache Camel	camel	Jul, 2009 to Apr, 2016	78	965
Tomcat Jasper	tomcat-jasper	Jul, 2010 to Jab, 2016	87	255
Apache Wicket	wicket-datetime	June, 2007 to Apr, 2016	110	17
Jetty SPDY	spdy-http-server	Sep, 2012 to Apr, 2016	52	47
Librato Metrics	metrics-librato	July, 2012 to Mar, 2016	34	17
Apache MyFaces	myfaces-impl	Jun, 2006 to Apr 2016	80	215

The rest of the paper is organized into eight sections. In section 2, we summarized the related work about the use of change impact analysis techniques and applicability of certain coupling metrics and tools. In section 3, we describe the structure of proposed approach HICSP. In section 4, we present our first study for empirical investigation to assess the effectiveness of HICSP. In section 5, we present our second replicated study to present the impact on the performance of HICSP, when the parameters of stability assessors are tuned. In sections 6 and 7, we formulate the results of both studies and present some implications for the researchers. Finally, in sections 8 and 9, we present some threats to the validity and the conclusion of our work respectively.

## Related work

We summarized and provide an overview of researcher’s previous efforts related to the existing approaches and structural coupling metrics. These techniques have been used to analyze and predict the classes prone to the ripple effect.

### Change impact analysis

Change Impact Analysis (CIA) techniques are applied to quantify the impact of changes across the parts of a system.

The implication of change impact analysis techniques are valuable for effort estimation [14], program comprehension [15], to prioritize the test cases, and exhibit the relationship among the components [16].

The term CIA is coined by Horowitz and Williamson [17]. However, in a recent review study, Li et al. [1] investigates 23 change impact analysis techniques. Subsequently, the authors of study [1] grouped the CIA techniques based on 1) the information collected from the execution of a program, 2) the information mined from software repositories, 3) the different types of coupling such as structural, conceptual, dynamic functional and relational, and 4) combining several change impact analysis techniques to present a hybrid approach. Ramanathan et al. [18] describes a tool namely Sieve which aims to detect the variation across the program versions by examining the execution of two binaries on the same test input and return the affected functions prone to changes. The experimental results suggest the applicability of Sieve to reduce the time and effort required for program testing and software maintenance. Law and Rothermel [19] focused on the limitation of three existing traditional dependency-based impact analysis techniques such as a call graph based analysis, static, and dynamic program slicing. In order to overcomethese issue, authors [19] present a whole path profiling based impact analysis technique. Subsequently, the proposed technique is based on the dynamic information obtained through simple program instrumentation and provides a different set of cost-benefits tradeoffs. The existing impact analysis techniques which based on dynamic information are either expensive in term of execution head or imprecision. However, in terms of precision and recall performance measure, Apiwattanapong et al. [20] present a new algorithm based technique which is more precise and efficient as compared to existing dynamic information based impact analysis techniques. The proposed technique collects the Execution-After sequence using a list of events, which is updated at each method entry and flow of control before control return to themethod call. Researchers have used historical data to understand programs and detect their evolution at certain granularity levels, such as coupling between files [21, 22], classes [23], to detect coupling between fine-grained program entities functions and variables [24]. The objective of version history based approaches is to extract the co-change coupling between the files, classes or functions for change impact analysis through the use of different data mining techniques, such as authors of study[24] uses data mining techniques to obtain association rules from the version histories of eight projects. Subsequently, in contrast to [21, 22, 23, 24], Torchiano and Ricca [25] used source code comments and changelogs in the software repository to extract the unstructured knowledge and support the change impact. Researchers have theoretically and empirically validated the use of metrics [8, 9, 10, 11, 12] to measure the certain types of coupling for the change impact analysis of program artifacts (such as classes) and the impact of static relationship between pattern and anti-pattern classes [26] on the quality attributes. These metrics aid to measure the structural coupling [11, 27, 28], dynamic coupling, evolutionary, logical coupling [29], and conceptual coupling [13, 30].

### Existing tools and coupling metrics for CIA

The decision regarding making changes in a software module (e.g. A class) is subject to the various factors which can aid to predict the stability of the corresponding module. The probability of future change in a class depends on the likelihood of changes occurs inside the class besides inherit changes which occur in other related classes. Tsantalis et al. [7] used the term ‘axis of change’ for the dependencies between the classes, which can produce change propagation from a source class to the dependent class(es). Subsequently, the authors analyzed the axes of change with its corresponding class and calculate the instability acquired by each axis of change. Black [31] reformulates the original ripple effect algorithm (produced by Yaun and Collofello in 1978) using matrix arithmetic, implement it in his existing automatic tool named REST (Ripple Effect and Stability Tool), and compare its performance with existing automatic tools named DPUTE (Data-centered Program Understanding Tool Environment), SMIT and ChAT to trace the ripple effect. The measurement of dependencies between the program entities is an ironic and stimulating body of work. Such measurement results in various coupling metrics, which can be considered as assessor of design stability of classes, such as Coupling Between Object (CBO) and Response For a Class (RFC) from Chidamber and Kemerer’s metrics suit [9, 11], Data Abstraction Coupling (DAC) and Measure of Aggregation (MOA) from QMOOD [10] and Message Passing Coupling (MPC) [12]. The aim of dependency analysis is to explore the ripple effect caused by a class change in the sense of change propagation across the system classes through their dependencies. The containment, generalization, and association are three common types of dependencies which can propagate changes across the system. Usually, structural coupling metrics used the method invocation and attributes referenced [13] to measure these propagated changes. For example, MPC and RFC measures based on method invocation only, whereas the CBO measure is based on both method invocation and attributes referenced.

In a recent study, Ampatzoglou et al. [5] considered these change propagation factors and used a new measure Ripple Effect Measure (REM) to investigate the effect of the Gang-of-Four (GoF) design patterns on stability. Moreover, in their subsequent study, Arvantou et al. [6] theoretically and empirically investigate the impact of the REM measure in order to address the issue of change propagation occurs inside source classes. These studies provide evidence that class stability is assessed through a source code snapshot using coupling and change propagation metrics as assessors. However, it is still not reported that whether the historical information regarding change propagation factors and their correlation can aid to predict the class instability. In this paper, we follow the methodology [6] and introduce a new approach Historical Information for Class Stability Prediction (HICSP), to exploit change history information and predict the instable classes based on its correlation with the change propagation factors.

## HICSP overview

The key idea behind Historical Information for Class Stability Prediction (HICSP) is to predict the instable classes through exploiting the historical information of change propagation factors (For example, number of polymorphic methods, method calls, and number of protected attributes in case of generalization, containment, and association). These propagation factors are mined from a desire versioning system. Firstly, HICSP extract information (needed to predict the instable classes) from the versioning system through a component called Change History Extractor. The information about the change propagation factors of a class in each version is collected using the tool Percerons Client (http://www.percerons.com/). In this study, we consider the number of polymorphic and total methods, protected attributes, and distinct method calls factors which can promote changes across the class relationships. Subsequently, the collected information and a classification algorithm are provided as an input to the stability assessor (component of HICSP) for the prediction of instable classes. In this paper, for each incorporated classifier, we used the term ‘Stability assessor’ and labelled with HICSP. For example, in section 4, we incorporate Random Forest (RF) classifier in the proposed approach HICSP and label it as HICSP+RF (stability assessor). We used same labelled convention for other incorporated classifiers (Section 4 for detail). The descriptions of components of HICSP are as follows.

### Change history extractor

This component of HICSP performs two tasks. The first task is to mined the desired versioning system (For example git, SVN or CVS) logs to report the entire change history of a system under analysis. This can be done by incorporating certain tools and repositories(http://search.maven.org/).

The logs which are extracted from the first task report the code changes at the file granularity level. The second task is performed in the context of the Percerons Client^1^ to analyze the change propagation factors and identify the class dependencies in a desired source code snapshot of a system. The Percerons Client provides a list of web services that aid developers to adopt different software engineering approaches in their product development. Currently, the services of Percerons are grouped into three categories, a) Percerons Reuse Repositories, b) Percerons Quality Dashboard, and c) Percerons Design Expert. Subsequently, Percerons Client performs these services using pattern-based and dependencies based search engines. These engins work at certain granularity levels such as attributes, methods, and classes. The extracted information is recorded into a dataset. The structure of a dataset is described in Table 2. The recorded information about the change propagation factors of class might be remained constant and consider as a threat to the performance of a stability assessor component of HICSP. Consequently, in order to improve the data quality by removing data inconsistency [32], we recommend some pre-processing activities which could be performed before the classification decisions through stability assessors.

**Table 2:** Dataset structure.

Variable	Description
V1-V3	These demographic variables are used to record project name, version, and class name respectively.
V4-V7	These independent variables are used to record the ripple change propagation factors, such a Number of polymorphic and total methods, protected attributes, and distinct method call in case of class relationships.
V8-11	These variables are used to record the REM, CBO, RFC and MPC values respectively.
V12	This dependent variable is labeled 1 if a change in class is encountered between two consecutive versions due to ripple effect otherwise 0.

### Stability assessor

The Change History Extractor component of HICSP is used to extract and record the version history of each class of a system (under study). The recorded information about a class together with a classification algorithm is used as an input to the Stability assessor component. The Stability assessor incorporates a classification algorithm with its default parameters and named as a customize stability assessor. However, the performance can be improved by tuning the parameters of incorporated algorithms (objective of second study). Subsequently, the aim of stability assessor is to predictinstable classes in a current source code snapshot. The decision of stability assessors about the instability of a class (in a current version) depends on their learning regarding the version history (i.e. Prior versions) of change propagation factors of a class. For example, a system under study is represented as a set of N snapshots (i.e. Versions) which is described as *S* = {*s*_1_,*s*_2_,…,*s*_*N*_}. Subsequently, each snapshot *s*∈*S* has a set of M classes which is described as *C* = {*c*_1_,*c*_2_,…,*c*_*M*_}. The historical information of a system under study is formulated as *S*×*C* = {(*s*_1_,*c*_1_),(*s*_1_,*c*_2_),…,(*s*_1_,*c*_*M*_),(*s*_2_,*c*_1_),…,(*s*_*N*_,*c*_*M*_)} and record in the dataset (Table 2). The decision of stability assessor regarding a class *c*_1_ in an ith snapshot *s*_*i*_∈*S* (i.e. Testing dataset) depends on its learning on its change propagation factors in the prior (i-1) versions {*s*_1_,*s*_2_,…,*s*_*i*−1_}∈*S* (i.e. Training dataset).

## Evaluation of HICSP (First study)

The purpose of this study is to evaluate the HICSP in order to identify classes highly affected by ripple changes (or in other words to predict instable classes). The evaluation process of HICSP is described in the following subsections.

### Context selection

The context of the study consists of the version history of 10 open source software projects named MongoDB Java Driver, Apache CXF, Apache Lucene, Google Guava, Apache Camel, Tomcat Jasper, Apache Wicket, Jetty SPDY, Librato Metrics and Apache MyFaces. We used git versioning system to mined the version history of the systems. The list of projects, corresponding artifactID, time slot, total versions, and the number of classes for each system are shown in Table 1. We collect version history of each project upto April, 2016. The brief description of selected projects is given as.

1. **MongoDB Java Driver:** MongoDB Java Driver help to provide synchronous and asynchronous interaction with MongoDB. It includes the legacy API (Application Programming Interface) and a new generic MongoCollection interface to compile new cross-driver CRUD (acronym for Create, Read, Update and Delete) specification.

2. **Apache CXF:** Apache CXF (as the product of two projects Celtix and XFire) is an open-source services framework which helps to build and develop services using JAX-WS (Java API for XML-Based Web Servies),JAX-RS (Java API for XML-Based RESTful Web Servies) APIs, and communicating protocols such as HTTP (Hype Text Transfer Protocol), SOAP(Simple Object Access Protocol), XML.HTTP, and CORBA (Common Object Request Broker Architecture).

3. **Apache Lucene**: Apache Lucene is an open source, high performance, and text-based search engine written in Java. The scalability, high-performance indexing, cross-platform solution, and efficient searching algorithm are the powerful features of Apache Lucene.

4. **Google Guava:** Google Guava is an open source set of libraries for Java to aid the developers. It includes 1) the utilities to reduce the menial labors to implement common methods and behavior, 2) An extended Java Collection Framework (JCF), and 3) the utilities to provide cache hashing and functional programming.

5. **Apache Camel:** Apache Camel is a mediator and rule-based routing engine which can aid for object-based implementation of Enterprise Integration Patterns through APIs based on a domain specific language. Apache ActiveMQ and Apache ServiceMix are Companion of Apache Camel in order to develop the SOA (Service Oriented Architecture) based projects.

6. **Tomcat Jasper:** Tomcat Jasper is a JSP Engine used to implement the Java Server Pages specification. It is developed and maintained by the Java Community Process. A Java Specification Request (JSR) is instantiated to start each specification.

7. **Apache Wicket:** Apache Wicket is a lightweight component based web application framework which is used throgh Java programming language. In contrast to traditional MVC (Model View Controller) frameworks, apache wicket is more close to Swing as stateful GUI framework in term of the whole request and response pages.

8. **Jetty SPDY:** SPDY (pronounced as speedy) is an open specification networking protocol developed by Google for transporting the web content and manipulating HTTP traffic. Jetty is a Java HTTP web server and servlet container which aid for the communication among machines of larger software frameworks. Subsequently, Jetty aid for a client and a server implementation of SPDY protocol using four modules spdy-core, spdy-jetty, spdy-jetty-http, and spdy-jetty-http-webapp modules.

9. **Librato Metrics:** Librato Metrics gem is used to provide granular control for scripting interactions with the Metrics core API. In Librato, a metric referred as a variable to measure the CPU load on different servers. Subsequently, this gem is well suited for the scripts, integrations workers, and background jobs.

10. **Apache MyFaces:** Apache software foundation introduces the project Apache MyFaces to host several JavaSever technologies related sub-projects. JavaServer is based on well-established standard MVC for the web based development frameworks in Java. Besides, Apache MyFaces provides several component libraries which contain UI widgets to develop the web-based application with JSF (JavaServer Faces), for example MyFaces Tomahawk and MyFaces Tobago.

In order to simulate the use of HICSP, we recorded the history of subject systems into a dataset structure depicted in Table 2.

### Research objective and research questions (RQs)

The existing structural coupling metrics are capable of measuring the relationship intensity of a class using method invocation and attributes referenced [13]. Subsequently, the metrics (such as REM) are used to measure the ripple effect of changes occurs in the dependent classes to analyze the stability of a source class. However, these metrics lack the ability to work in the capacity to use the historical information regarding change propagation factors of a class, and predict whether it is prone to ripple effect (i.e. Instable class) or not (i.e. Stable class). The primary objective of our study is to introduce and implement HICSP in order to predict the instable classes which are more prone to the ripple effect (changes occur in their dependent classes). Subsequently, we evaluate the HICSP’s performance as compared to existing coupling and ripple effect measures (recommended as stability assessors). Finally, in our replicated study, we evaluate the effect of parameters tuning on the effectiveness of HICSP. Through our objective statement, we extract and formulate three research questions.

**RQ-1.** How does HICSP perform in predicting the instable classes?

**RQ-2.** How HICSP outperform as compared to other class stability assessors.

**RQ-3.** How the performance of HICSP is influenced when the parameters of incorporated classifier are tuned.

We respond to RQ-1 and RQ-2 in our first study, while in the second study, we respond to RQ-3.

### Classifiers incorporated with HICSP

Recently, MDelgado et al. [33] have evaluated the performance of 179 classifiers of 17 different families on 121 datasets (retrieved from the UCI database) to solve the real world classification problem. In their study, the authors concluded the variation in the performance of classifiers. However, Random Forests (RF), Support Vector Machines (SVM), C4.5 Decision Tree and Naïve Bayes are reported comparatively better than other classifiers. We used WEKA (Waikato Environment for Knowledge Analysis) an open source machine learning tool (http://www.cs.waikato.ac.nz/ml/weka/) and select four classifiers named Random Forest, SMO (an alternative of SVM in Weka), J48 (an alternative of C4.5) and Naïve Bayes. We incorporate these classifiers (with default parameters) in the stability assessor component of HICSP. Moreover, we labelled these classifiers as HICSP+RF. HICSP+SMO, HICSP+J48, and HICSP+NB. Numerous researchers have used these classifiers and report their effectiveness regarding classification decisions. For example, Catal and Diri [32] and Ma et al. [34] reported that Random Forests classification algorithm provides the best performance in terms of F-measure (evaluation parameter). Random Forest algorithm [35] implements tens or hundreds of trees depends on dataset characteristics and later use the results of these trees for the classification. Subsequently, Menzies et al. [36] stated that Naïve Bayes achieves better performance (especially with logNums filter) in terms of the probability of detection (pd) and probability of false alarm (pf) values. The brief description of selected classification algorithms is given as:

1. **Random Forests:** Random Forest classification algorithm implements tens or hundreds of trees depends on the dataset characteristics and later use the results of these trees for the classification. In their study, Koprinska et al. [35] stated that Random Forest is a good choice for filing emails into corresponding folders and filtering of spam email. The authors reported the better performance of RF as compared to decision trees and Naïve Bayes, especially in terms of F-measure [32, 34]. Subsequently, Random Forest builds each classification tree using a bootstrap sample of the dataset and the candidate set includes randomly selected variables at each split [37, 38]. The best split based on the randomly selected variables in the training set.

2. **J48:** J48 is a decision tree which based on Quinlan’s C4.5 algorithm [39] and implemented in Weka tool. The Australian Research Council has funded to conduct research on the C4.5 algorithm and introduce it as a well-known machine learning algorithm. The notation of J48 decision tree includes internal nodes, branches, and terminal nodes which represent the attributes, values associated with attributes, and classification results respectively.

3. **Naïve Bayes:** Naïve Bayes is a well-known probabilistic classifier based on the Bayes theorem. Naïve Bayes classifier is implemented with strong independence assumptions, which mean the existence of a class’s feature does not depend on the existence of the other features. The Naïve Bayes classifier can estimate the parameters with small data quantity [40, 41, 42].

4. **SMO:** Sequential Minimal Optimization (SMO) is an efficient classification algorithm implemented in Weka for training the support vector machines. Usually, support vector machine (SVM) is trained with a solution of a large Quadratic Programming (QP) optimization problem. Sequential Minimal Optimization (SMO) algorithm is used to solve the SVM quadratic problem without considering extra matrix storage and using numerical QP optimization procedures [43].

The adjustment of parameter values of these classifiers (due to the existence of noise in the dataset) can aid to improve the effectiveness of HICSP. We have summarized the list of certain parameters of selected classifiers in Table 3, which can be used to replicate the proposed study.

**Table 3:** Summary of parameters for stability assessors.

Stabilityassessors	Parameter	DefaultValue	Description
Random Forest	Number of Iteration	100	This parameter is used to tune the number of trees in aconstructed forest.
	Number of Attributes	0	This parameter is used to tune the number of features for aconstructed forest according to log2(M)+1.
	Number of slots for execution	1	This parameter is used to auto tune the number of slotswith respect to max heap size.
J48	Confidence Factor	0.25	This parameter is used to tune the effectiveness of a postpruning method.
	Number of Instances	2	This parameter is used to tune the number of instances forthe effectiveness of the on-line pruning method.
NaïveBayes	Type of Estimator	NormalDistribution	This parameter is used to tune the model through a kerneldensity estimator rather than a normal distribution.
SMO	Complexity level	1	The high skewed data and overlap classes lead to thelonger time for the training. This parameter is tuned withrespect to data skewness and class overlapping.
	Normalization	0	The normalized data aid to reduce training time. This parameter is tuned for normalization of training data.
	Internal Cross-Validation	-1	This parameter is used to tune the number of folds forinternal validation of the constructed model.
	Number of Iterations	-1	This parameter is used to tune the number of iterations

### Experiments procedure

We record the version history of each system with N snapshots and M classes (Table 1) into dataset (Table 2). Subsequently, in order to maintain accuracy in the obtained results, for each subject system, we record the version history of only those classes which are found in the first and a random snapshot selected for the analysis. The refactor and rename classes are not included in the analysis. In order to perform each experiment, we used the same procedure described as follows.

**Step-1:** Select the system S with N versions (i.e. Snapshots).

**Setp-2:** Retrieve the M classes exists in all versions of S.

**Setp-3:** Select the ^i^th version randomly from N versions to predict the instable classes.

**Step-4:** Select a class from the ^i^th version.

**Step-5:** In order to predict stability of the selected class, we train the stability assessors on (i-1) prior versions of the selected class.

**Step-6:** Repeat Step-4 and Step-5 for all classes.

**Fig 3:**
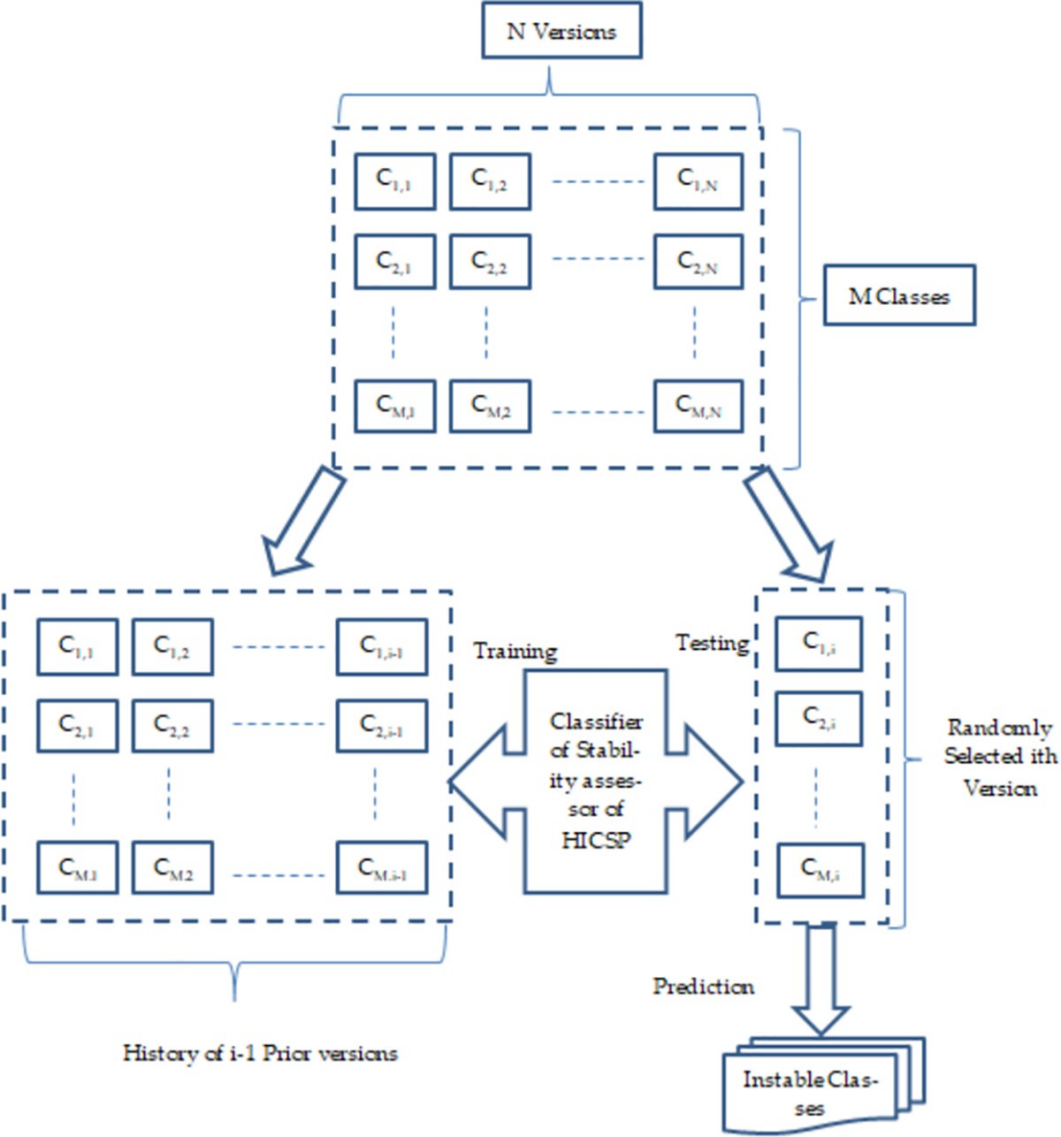
Working procedure of stability assessors in experiments.

Subsequently, in each experiment, the training and testing procedure is described in Fig 3. The classifier’s decision regarding the instability of a class (in ^i^th version) depends on its learning on the version history (i-1 prior versions) of propagation factors (associated with the corresponding class).

### Evaluation criteria for HICSP

In a recent study, Tantithamthavorn et al. [44] conduct an empirical study to empirically investigate the bias and variance of existing validation techniques which are used for the prediction models especially in the domain of defect prediction. In their study, author [44] reported that 49% studies have used of k-fold cross-validation to obtain results which are less prone to bias and variance. Consequently, we performed k-fold (i.e. k = 10) cross-validation for the precise learning of stability assessors on the training dataset. In order to evaluate the capacity of each stability assessor, we create a confusion matrix which includes True Positive (TP), True Negative (TN), False Positive (FP), and False Negative (FN).

1. A class will be counted as True Positive (TP) if it is prone to ripple effect in reality and through the corresponding stability assessor it is also predicted as prone to the ripple effect.

2. A class will be counted as True Negative (TN) if it is not prone to the ripple effect in reality and through the corresponding stability assessor it is also not predicted as prone to the ripple effect.

3. A class will be counted as True Positive (FP) if it is prone to the ripple effect in reality and through the corresponding stability assessor it is not predicted as prone to the ripple effect.

4. A class will be counted as True Negative (FN) if it is not prone to the ripple effect in reality and through the corresponding stability assessor it is predicted prone to the ripple effect.

Subsequently, we used F-measure as an aggregate indicator harmonic mean of two widely adopted Information Retrieval (IR) metrics that is Precision and Recall [18]. The recall, precision, and F-measure are used to evaluate the performance of stability assessors, shown in Eqs 1–3 respectively.

Recall=TP/(TP+FN) (1)

Precision=TP/(TP+FP) (2)

F‐measure=2×(Precision×Recall)/(Precision+Recall) (3)

### Result discussion on effectiveness of HICSP

**Respond to RQ-1:**In order to respond RQ-1, we performed experiments by using the procedure described in the Section 4.4. For example, we record the history of MongoDB Java Driver with 88 versions (i.e. N = 88) and 268 classes (i.e. M = 268). Subsequently, we randomly select an ith git snapshot (i.e. e34e979) committed on 18, November 2015. We train the stability assessors on all prior snapshots delivered before November 2015 and evaluate their performance on the ith snapshot (i.e. e34e979). We evaluate the capacity of stability assessors using criteria described in Section 4.5 to predict the instable classes in the corresponding snapshot. In this regard, the main consequences are;

- The performance of each stability assessor varies across the projects due to differences in dataset characteristics such as the performance of HICSP+RF remain highest for Apache Camel and Tomcat Jasper (F-measure = 93%) as compared to rest of projects.
- We cannot recommend that all statbility assessors work same for a single dataset, which indicate the variation in influence of discriminative power of stability assessors, for example, for Apache CXF project, the performance of HICSP+RF(F-measure = 85%), HICSP+J48(F-measure = 82%), HICSP+SMO(F-measure = 80%), and HICSP+NB(F-measure = 81%) varies.
- Finally, we cannot benchmark the performance of stability assessors due to differences in their performance across the projects. Consequently, we need to use of non-parametric tests to benchmark their performance and recommend an outperformed stability assessor.

The comparative performance of stability assessors (HICSP+RF, HICSP+NB, HICSP+SMO, and HICSP+J48) in term of recall, precision and F-measure values is shown in Table 4. Since the F-measure values achieved by HICSP+RF on all datasets is better with minor difference as compared to other stability assessors HICSP+J48, HICSP+SMO and HICSP+NB. Consequently, we perform three non-parametric Friedman, Nemenyi, and Analysis of Means (ANOM) tests to benchmark the outperform stability assessor. Firstly, we apply Friedman’s Test on the F-measure values of each stability assessor to achieve the chi-square at p-value = 0.05. The Friedman’s Test chi-square value 59.89 is greater than the critical value 7.68 with the degree of freedom (df) as 1, which suggest the rejection of the corresponding null hypothesis (H0: Stability assessors are evenly performed on all datasets). Consequently, we can report that there is a significant difference between the F-measure values of each stability assessor.

**Table 4:** Performance evaluation of stability assessors (HICSP with incorporated classifiers) in terms of f-measure.

Projects	HICSP+RF			HICSP+J48			HICSP+SMO			HICSP+NB		
	R	P	F	R	P	F	R	P	F	R	P	F
MongoDB Java Driver	80	82	81	78	80	79	75	76	76	78	79	79
Apache CXF	83	87	85	79	84	82	76	80	78	79	83	81
Apache Lucene	85	88	87	84	86	85	83	84	84	75	80	78
Google Guava	87	95	91	85	90	88	87	90	89	85	88	87
Apache Camel	90	96	93	88	92	90	87	89	88	80	88	84
Tomcat Jasper	91	95	93	90	94	92	84	89	87	79	85	82
Apache Wicket	89	94	92	82	89	86	84	90	87	81	84	83
Jetty SPDY	90	94	92	92	92	92	86	88	87	87	88	88
Librato Metrics	81	89	85	84	86	85	81	80	81	79	83	81
Apache MyFaces	86	90	88	82	86	84	80	78	79	80	79	80

**Fig 4:**
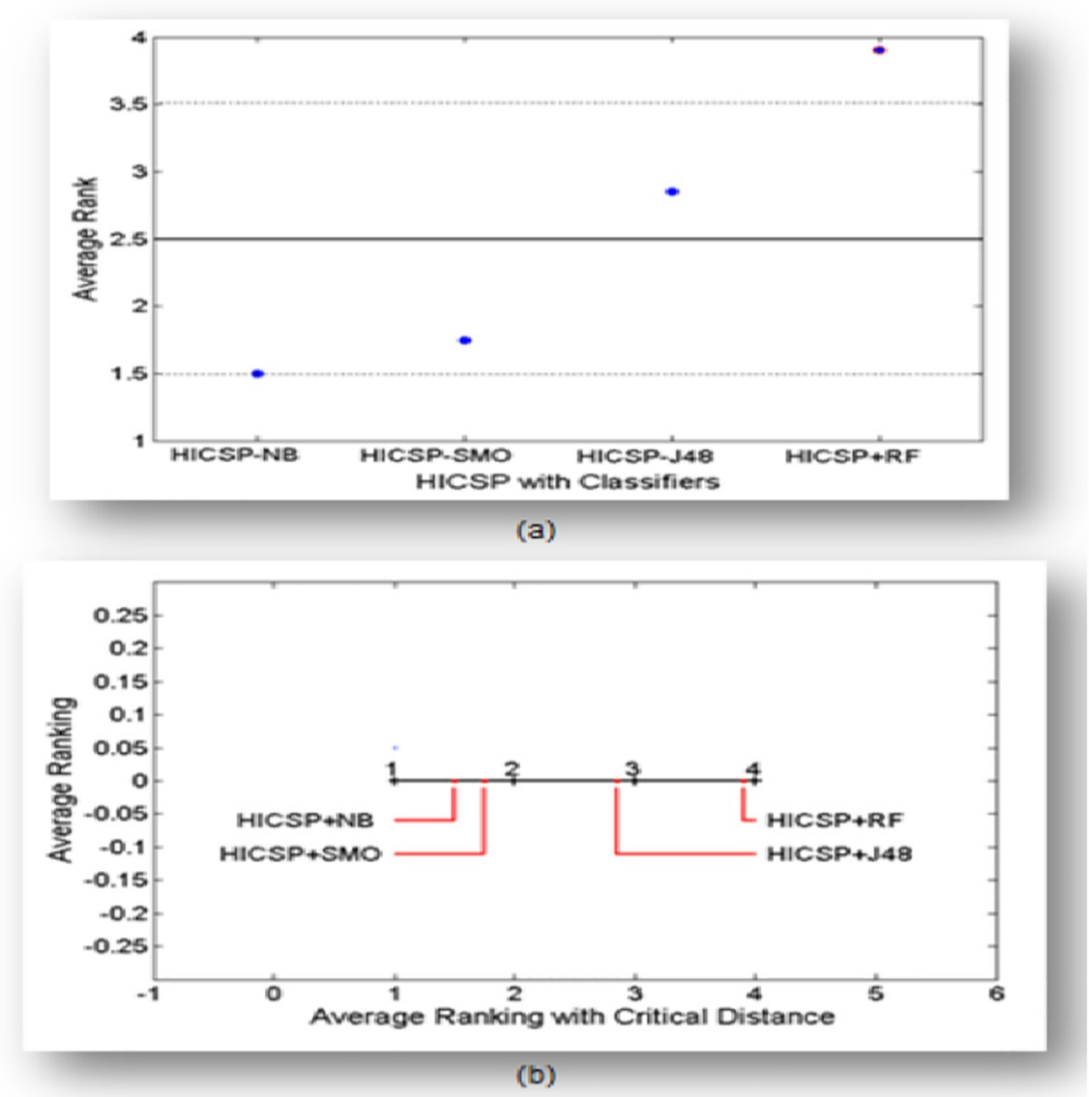
ANOM and Nemenyi tests for stability assessors. a) ANOM test result b) Nemenyi test result.

Secondly, we apply post-hoc Nemenyi and ANOM tests on the F-measure values of each stability assessor to compare their performance and rank them accordingly. The ANOM and post-hoc Nemenyi test’s ranking results are shown in Fig 4A and 4B respectively. The ranking of each stability assessor outside the limits presents their performance significantly worse than the average of all other competitive stability assessors.

The results of Table 4 and Fig 4 suggest the applicability of HICSP to predict the instable classes especially when Random Forest is incorporated (i.e. HICSP+RF).

**Respond to RQ-2:** In order to respond RQ-2, we consider the CBO, RFC, REM and MPC stability assessors in the analysis. We record the values of selected stability assessors for each version of a system into a dataset (Table 2). In order to evaluate the capacity of each stability assessor, again, we create a confusion matrix which includes True Positive (TP), True Negative (TN), False Positive (FP) and False Negative (FN). We execute CBO, RFC, REM and MPC stability assessors on the randomly selected ith snapshot (For example e34e979 in case of the MongDB Java Driver) to predict the instable classes and compare with outperform stability assessor (i.e. HICSP+RF) reported in the response of RQ-1 (Table 4). The comparison results in term of F-measure values are shown in Table 5. In this regard, main consequences are;

**Table 5:** Performance evaluation of outperform stability assessor (HICSP+RF) besides other stability assessors in terms of f-measure.

Projects	HICSP+RF	CBO	MPC	REM	RFC
MongoDB Java Driver	81%	76%	75%	79%	77%
Apache CXF	85%	77%	75%	81%	74%
Apache Lucene	87%	80%	79%	81%	77%
Google Guava	91%	81%	82%	84%	82%
Apache Camel	93%	80%	82%	90%	80%
Tomcat Jasper	93%	83%	80%	87%	82%
Apache Wicket	92%	83%	82%	87%	81%
Jetty SPDY	92%	84%	82%	85%	83%
Librato Metrics	85%	78%	78%	82%	78%
Apache MyFaces	88%	82%	81%	85%	81%

- Like state of the art stability assessors, the proposed approach HICSP is effective in terms of predicting the stable classes, such as the performance of HICSP-RF remain greater than 80% across the all projects.
- Due to minor differences in their performance, we cannot benchmark the performance of HICSP. Consequently, no-parameteric tests are required to benchmark the performance of HICSP.

**Fig 5:**
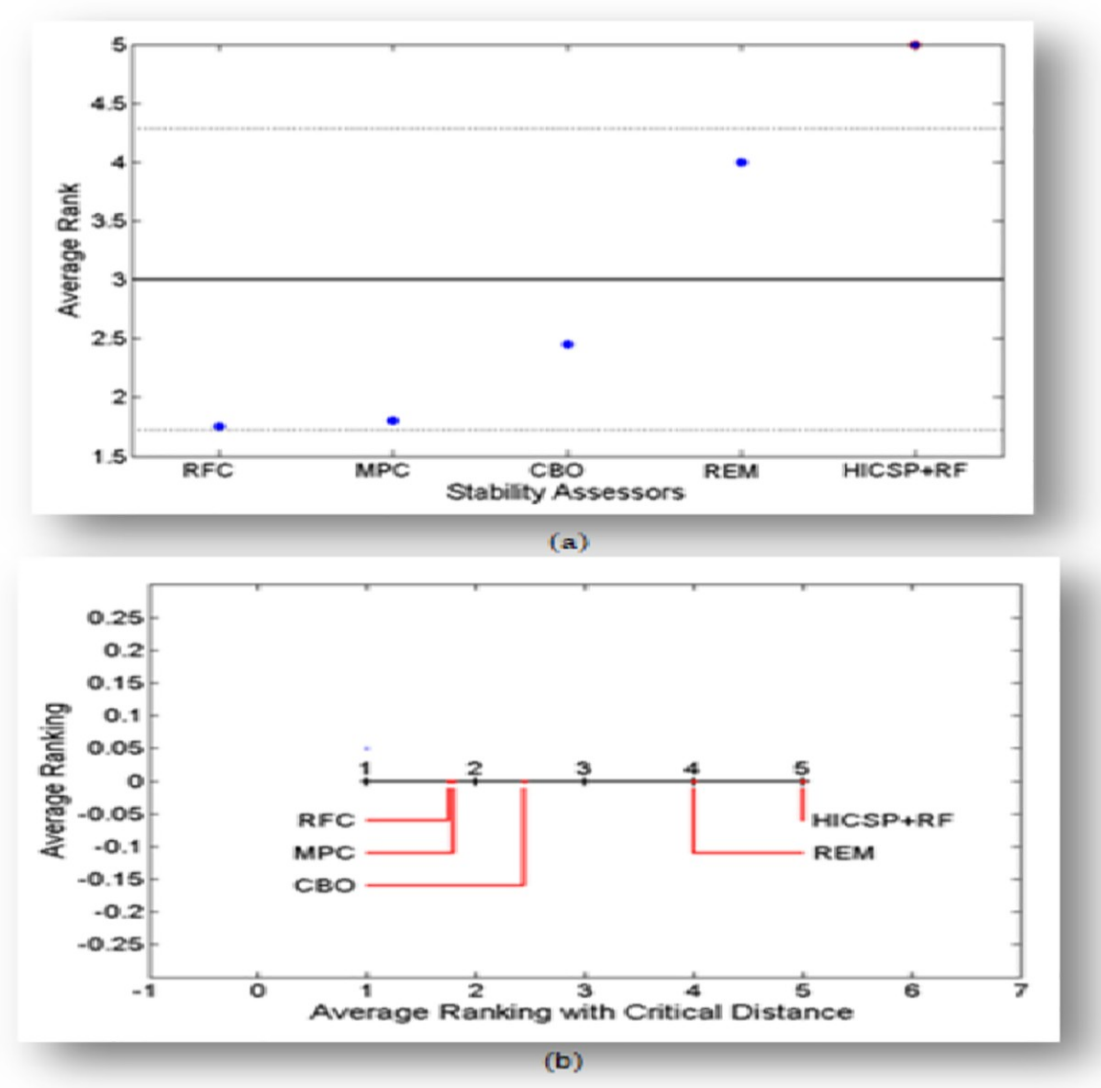
ANOM and Nemenyi tests for stability assessor (HICSP+RF) and stability assessors. a) ANOM test result b) Nemenyi test result.

The F-measure achieved by HICSP+RF on all datasets is better with minor difference as compared to other stability assessors. Consequently, we perform three non-parametric Friedman, Nemenyi, and ANOM tests to benchmark the performance ourperform stability assessor. Firstly, we apply Friedman’s Test on the F-measure values of each stability assessor to achieve the chi-square at p-value = 0.05. The Friedman’s Test chi-square value 78.18 is greater than the critical value 12.44 with the degree of freedom (df) as 1, which suggest the rejection of the corresponding null hypothesis (H0: Stability Assessors are evenly performed on all dataset). Consequently, we can report that there is a significant difference on the achieved F-measure value of each stability assessor. Secondly, we apply post-hoc Nemenyi and ANOM tests on the achieved F-measure of each assessor in order to compare their performance and rank them accordingly. The ANOM and post-hoc Nemenyi test’s ranking results are shown in Fig 5A and 5B respectively. The ranking of stability assessors outside the limits presents their performance significantly worse than the average of all other competitive assessors. The results of Table 5 and Fig 5 suggest that stability assessor HICSP+RF outperform than other stability assessors.

## Replicated study

We replicate the first study in order to evaluate the impact of tuning the incorporated classifier’s parameters (in the stability assessor component of HICSP) on the effectiveness of proposed approach HICSP. Though, we have summarized the list of parameters related to the incorporated classifiers (Section 4.3) and shown in Table 3, and performed certain experiments. However, in this replicated study, we only present stability assessor HICSP+J48 and investigate the effect of paramter’s tuning. We tune the confidence factor (i.e. for post-pruning) and minimum number of instances (i.e. for on-line pruning) of HICSP+J48, and apllied on the version history of MongoDB Java Driver open source project. In our first study, we have evaluated the effectiveness of HICSP+j48 with default value 0.25 and 2 of Confidence factor and minimum number of instances respectively. However, in this study, we performed a series of experiments by tuning these parameters and follow the same experimental procedure (mention in Section 4.4) and evaluation creteria (mention in Section 4.5).

### Respond to RQ-3

In order to respond RQ-3, we comparatively investigate the performance of HICSP+J48 with default and tuned values of Confidence Factor and Minimum Number of Instances parameters. The brief description of these parameters is as follows.

### Tuning of confidence factor

The confidence factor parameter is used to describe the effectiveness of post-pruning (i.e as pruning method) of j48. The post-pruning method in the C4.5 algorithm is regarded as the process of evaluating the decision error at each decision junction and its propagation. The decision error is computed through the Eq 4.

E=(e+1)/(N+m) (4)

**Fig 6:**
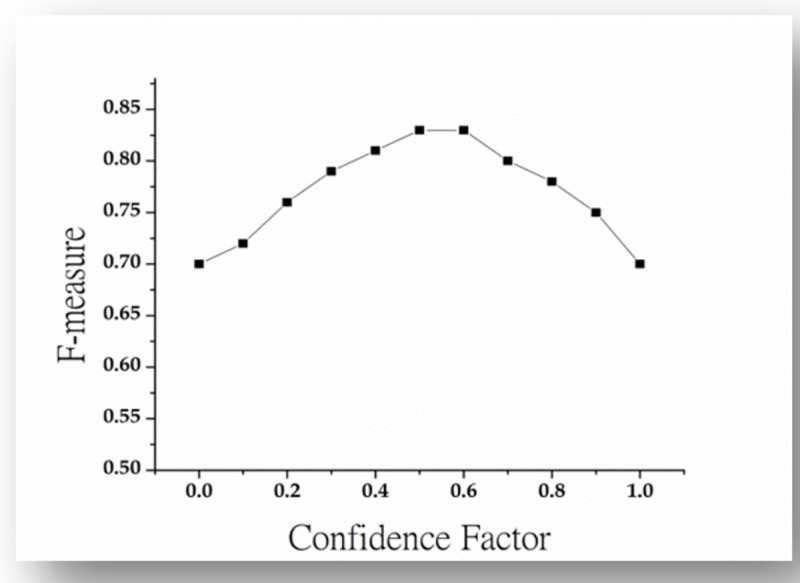
Performance evaluation of HICSP+J48 by tuning confidence factor parameter.

In the Eq 4, the term E, e, N and m referred as estimated error, misclassified instances, correctly classified and total instance of the given node. We performed experiments by ranging Confidence factor from 0.1 to 1.0, and keep the value 2 fixed for the minimum number of instance parameter of HICSP+j48. Subsequently, we performed each experiment with k-fold (i.e. k = 10) cross validation. The experimental results are summarized and depicted in Fig 6. The results of Fig 6 indicate that learning performance (in term of F-measure) of HICSP+j48 on the training dataset increases upto 83% when confidence factor ranges from 0 to 0.5, however, by increasing confidence factor from 0.5 to onward, the performance of HICSP+j48 decreases, which might be the cause of over-training.

### Tuning of minimum number of instances

**Fig 7:**
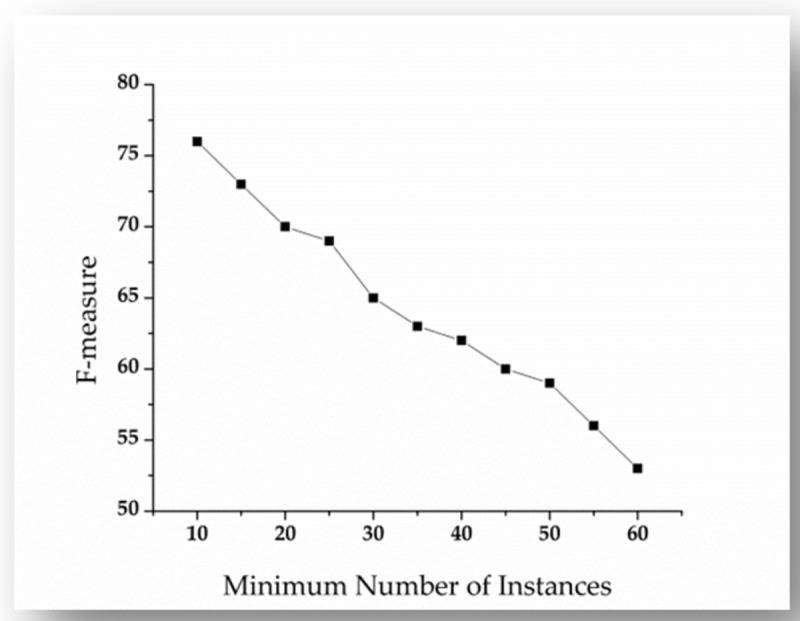
Performance evaluation of HICSP+J48 by tuning minimum number of instances parameter.

The minimum number of instance parameter of HICSP+J48 is used to describe the effectiveness of on-line pruning (i.e pruning method). The on-line pruning worked when decision tree is induced. The existing algorithms aid to divide the dataset on the attributes and provide information gain related to a class label. During the division process, a child’s leaf represents less than a minimum number of instances of a dataset. Subsequently, the parent and child nodes are combined and produce a single node. The compressing remains continue until the entire tree is created. We performed experiments by ranging minimum number of instances from 10 to 60, and keep the value 0.25 fixed for confidence factor parameter of HICSP+j48. Subsequently, we performed each experiment with k-fold (i.e. k = 10) cross validation. The experimental results are summarized and depicted in Fig 7. The results of Fig 7 indicate that the learning performance (in term of F-measure) of HICSP+j48 on the training dataset decreases to 62% when the confidence factor ranges from 10 to 40. Subsequently, an increase in the number of minimum instances causes to increase the over-training error.

## Results and discussion

### First study

The results shown in Table 4 and Table 5 suggest the applicability of proposed approach HICSP to predict the instable classes. In order to respond RQ1, the results (Table 4) of our first study indicate that HICSP’s precision (When RF, J48, SMO, and NB are incorporated) is between 75 to 92 percent and its recall is between 76 to 96 percent. The best classifier incorporated with proposed approach HICSP is evaluated in term of F-measure. For example, the prediction accuracy of stability assessor HICSP+RF is between 81 to 93 percent which remained better than the prediction accuracy of other stability assessors. Subsequently, in order to respond RQ2, we compare the performance of outperform stability assessor that is HICSP+ RF with existing stability assessors CBO, MPC, REM, and RFC. We used recall, precision, and F-measure to compare and evaluate the prediction accuracy of all stability assessors. The F-measure values achieved by HICSP+RF are between 81 to 93 percent on 10 systems, which is comparatively better than the achieved F-measure values of CBO (76 to 84 percent), MPC (75 to 82 percent), REM (79 to 90 percent) and RFC (74 to 83 percent). In the term of F-measure, on some systems we did not find any significance difference between HICSP+RF and other stability assessors. For example, on MongoDB Java Driver, Apache CXF, Librato Metrics and Apache MyFaces datasets, there is the minor difference between the prediction accuracy of HICSP+RF and REM stability assessor.

### Second study

In the second study, we consider only HICSP+J48 and investigate the effect of tuning the two parameters Confidence Factor and Minimum Number of Instances on its effectiveness with MongoDB Java Driver dataset. The result shown in Fig 6 and Fig 7, which indicates the significant increase (in term of F-measure) in the performance of HICSP+J48. For example, in case of HICSP+J48 with default parameter values (Confidence Factor as 0.25 and Minimum Number of Instances as 2), we have observed the performance 79% (shown in Table 4). From the results shown in Fig 6, we observed a 5.06% increase in the performance of HICSP+J48 at the value 0.50 of confidence factor. Subsequently, from the results of Fig 7, we can observe a significant decrease in the performance of HICSP+J48, when the minimum number of instances are increased.

## Implication for researchers

Though, in this study, we exploit and model the evolution history of change propagation factors in order to predict the instable classes. However, we recommend certain dimensions for researchers about the use of the proposed approach.

In this study, we cannot examine the explanatory variable (i.e. Change propagation factors) individually in the relation to the class instability. The proposed approach can be used to examine the influence of each propagation factor throughout the evolution of a class and predict the highly influential (i.e. Hotspot) change propagation factor. This will aid maintainers to reduce the time and efforts required to maintain the class and to understand certain aspects of instable classes.

The proposed approach can be used to investigate the differences between pattern, anti-pattern, and pattern classes associated with at least one anti-pattern class in term of their stability. For example, the pattern classes are more stable than those pattern classes which are associated with at least one anti-pattern class [26].

The proposed approach can be used to compare the quality differences between software libraries and standalone applications, because in case of software libraries it is generally believe that developers of software libraries followed the design guidelines due to need of continuous evolution.

## Threats to validity

In our study, we also find some threats. The first threat is related to external validity to generalize the results. The HICSP is evaluated with the version history of only ten projects. The prediction accuracy of HICSP can be analyzed to consider the long version history projects such as Apache OpenOffice in the study. Subsequently, the incorporation of more classification algorithms can present the variation in the performance results of HICSP. The second threat is related to the internal validity of factors, which could influence the results. We incorporate the classification algorithms with default parameters adjusted in Weka tool. However, the calibration of parameters of classification algorithms could improve the prediction accuracy of HICSP. Though, in the second study, we have empirically investigated the effect of tuned parameters (i.e Confidence Factor and Minimum Number of Instances) on the performance of HICSP+J48. However, we need to tune the parameters of other stability assessors in order to generalize the results.

## Concluion and future work

We presented HICSP (Historical Information for Class Stability Prediction), an approach aimed to predict the instable classes by exploiting history information of change propagation factors extracted from the versioning systems. In order to respond to our research questions, we conducted two empirical studies. The promising results are produced from both studies, which can suggest the developers to apply the HICSP in order to predict the instable classes (in terms of promoting the ripple effect) through the analyses of version history of its change propagation factors, such as a number of polymorphic methods, the number of protected attributes, and distinct methods calls. We incorporate four widely-used classification algorithms Random Forest (RF), J48, Sequential Minimal Optimization (SMO) as a variant of Support Vector Machine (SVM), and Naïve Bayes (NB) in the proposed approach HICSP as stability assessors and evaluate its prediction accuracy. In terms of precision, recall, and F-measure, we observed the significance prediction accuracy of HICSP especially when RF is incorporated. Such as, the performance of HICSP is reported in range from 81% to 93% across the all projects. Subsequently, in comparison with existing design class stability assessors REM, CBO, MPC, and RFC, we observe 4 to 16 percent (in term of F-measure) improvement in the prediction of instable classes. However, it can be improved by tuning the parameters to overcome the noise in the training data. For example, we observe the performance of HICSP+J48 as 79% in terms of F-measure with default parameters values (Confidence Factor = 0.25 and Number of Instancess = 2) on MongoDB Java Driver dataset. By tuning the Confidence Factor (from 0 to 0.50), we obervee approximaltely 5% (F-measure) increase in the performance. We are planning to incorporate Bayesian Vector Auto Regression (Bayesian VAR) with the proposed approach to estimate the stability of a class through interdependencies of change propagation factors and model the stability of a whole system.

## Supporting information

S1 File Datasets used in study. (https://drive.google.com/file/d/1TeV6pDRGVggT3uzFmfkhMPBlbiz6IlUJ/view?usp=sharing)
(RAR)

## Abbreviations

CIA: Change Impact Analysis
REM: Ripple Effect Measure
CBO: Coupling Between Object
RFC: Response For a Class
DAC: Data Abstraction Coupling
MOA: Measure of Aggregation
MPC: Message Passing Coupling
HICSP: Historical Information for Class Stability Prediction
GoF: Gang-of-Four
RF: Random Forest
NB: Naïve Bayes
SMO: Sequential Minimal Optimization
Pd: probability of detection
Pf: probability of false alarm
TP: True Positive
TN: True Negative
FP: False Positive
FN: False Negative
ANOM: Analysis of Means
E: Estimated Error
E: misclassified instances
N: correctly classified
M: Total Instances
